# Open-CSAM, a new tool for semi-automated analysis of myofiber cross-sectional area in regenerating adult skeletal muscle

**DOI:** 10.1186/s13395-018-0186-6

**Published:** 2019-01-08

**Authors:** Thibaut Desgeorges, Sophie Liot, Solene Lyon, Jessica Bouvière, Alix Kemmel, Aurélie Trignol, David Rousseau, Bruno Chapuis, Julien Gondin, Rémi Mounier, Bénédicte Chazaud, Gaëtan Juban

**Affiliations:** 1 Institut NeuroMyoGène, Univ Lyon, Université Claude Bernard Lyon 1, CNRS UMR 5310, INSERM U1217, 8 Avenue Rockfeller, F-69008 Lyon, France; 2 CREATIS, Univ Lyon, Université Claude Bernard Lyon 1, CNRS UMR 5220, INSERM U1206, INSA Lyon, 69100 Villeurbanne, France; 3 CIQLE, Lyon Bio Image, Univ Lyon, Université Claude Bernard Lyon 1, Structure Fédérative de Recherche santé Lyon-Est CNRS UMS3453/INSERM US7, 69008 Lyon, France

## Abstract

Adult skeletal muscle is capable of complete regeneration after an acute injury. The main parameter studied to assess muscle regeneration efficacy is the cross-sectional area (CSA) of the myofibers as myofiber size correlates with muscle force. CSA analysis can be time-consuming and may trigger variability in the results when performed manually. This is why programs were developed to completely automate the analysis of the CSA, such as SMASH, MyoVision, or MuscleJ softwares. Although these softwares are efficient to measure CSA on normal or hypertrophic/atrophic muscle, they fail to efficiently measure CSA on regenerating muscles. We developed Open-CSAM, an ImageJ macro, to perform a high throughput semi-automated analysis of CSA on skeletal muscle from various experimental conditions. The macro allows the experimenter to adjust the analysis and correct the mistakes done by the automation, which is not possible with fully automated programs. We showed that Open-CSAM was more accurate to measure CSA in regenerating and dystrophic muscles as compared with SMASH, MyoVision, and MuscleJ softwares and that the inter-experimenter variability was negligible. We also showed that, to obtain a representative CSA measurement, it was necessary to analyze the whole muscle section and not randomly selected pictures, a process that was easily and accurately be performed using Open-CSAM. To conclude, we show here an easy and experimenter-controlled tool to measure CSA in muscles from any experimental condition, including regenerating muscle.

**Electronic supplementary material:**

The online version of this article (10.1186/s13395-018-0186-6) contains supplementary material, which is available to authorized users.

## Background

Skeletal muscle is capable of complete regeneration after an acute injury [1]. Skeletal muscle integrity is usually assessed by histological analyses, consisting of staining and/or immunofluorescent labeling of various cellular or molecular structural components of the tissue. Among these analyses, myofiber cross-sectional area (CSA) is widely used because it reflects the regenerative capacity of the muscle, i.e., the formation of new myofibers from the activation, proliferation, differentiation, and fusion of muscle stem cells [1]. CSA measurement is usually performed after immunofluorescent staining of laminin, a component of the basal lamina surrounding each myofiber, or of dystrophin that is located at the inside face of the sarcolemma.

Analysis methods range from manual quantification to programs showing various levels of automation. Manual quantification is potentially the most accurate method as the experimenter keeps total control over the myofibers being analyzed. However, the manual procedure is highly time-consuming and may present variability between experimenters. This is why automated programs were developed to limit the experimenter input and save time [2–7]. For example, SMASH [7] is a semi-automated open source MATLAB script allowing the assessment of several parameters, including CSA. MyoVision, a Windows program [2], was developed to fully automate the quantification process. This program combines several algorithms that were previously used separately in other programs, thus reducing the errors in the fiber identification and leading to a more accurate CSA measurement compared to SMASH on uninjured muscle [2]. Recently, MuscleJ, a fully automated plug-in in ImageJ described the fully automated quantification of various skeletal muscle parameters, including CSA [8].

Most of the automated softwares were tested on normal muscle or under conditions triggering atrophy/hypertrophy. In this context, the shape of the myofibers keeps polygonal and angular, myofibers keeping their contact with each other. On the contrary, regenerating muscle is characterized by the presence of round-shaped regenerating myofibers of highly variable size that, for the smallest ones, do not regularly contact surrounding fibers. Thus, variable myofiber shape, size, and extrafiber space characterize the regenerating muscle, rendering the automated quantification more difficult.

Here, we present a semi-automated CSA quantification method for skeletal muscle images applicable on any type of muscle and under any condition, including early and late regenerating muscle and dystrophic muscle. This method, named Open-CSAM (for Open [free]-Cross Sectional Area Measurement), is based on an ImageJ macro designed to automatically quantify CSA on immunofluorescent picture of the whole skeletal muscle section. It also includes some level of flexibility for the experimenter, allowing manual correction of the mistakes made by the automation. Moreover, it allows the analysis of the size of the myofibers on the whole muscle section, which we show here to be necessary to obtain an accurate measurement.

## Methods

### Animals and tissue preparation

C57BL/6 and mdx mice were used according to the French legislation. All experiments were performed on mice between 8- and 12-week-old, except for old mice, which were 2-year-old. In injury experiments, *Tibialis Anterior* (TA) muscles were injected with 50 μl of cardiotoxin (Latoxan) at 12 μM as previously described [9]. Fibrotic dystrophic mice (Fib-mdx) were generated as previously described [10] and mice were analyzed 1 week after the last injuries. TA muscles were mounted on pieces of cork and fixed with tragacanth gum. Then, they were frozen in isopentane cooled by liquid nitrogen and further stored at − 80 °C. TA muscles were sectioned thanks to a cryomicrotome (CM3050s, Leica), and the thickness of cryosection was 8 to 10 μm. The slides were then stored at − 80 °C until immunostaining. TA muscles showing more than 15% of uninjured area (i.e., without centrally located nuclei), indicating that CTX did not spread into the entire muscle, were excluded from the study.

### Immunofluorescence

Slides were dried for 10 min at room temperature. Muscle cryosections were encircled with a hydrophobic pen (Dako) and were incubated with PBS containing Triton 0.5% for 10 min and then washed three times with PBS. They were incubated with BSA 2% for 1 h at room temperature and then incubated overnight with a rabbit anti-laminin antibody (1:200, L9393, Sigma-Aldrich) at 4 °C in a moist chamber. Slides were washed three times with PBS and incubated with FITC-conjugated donkey anti-rabbit secondary antibody (1:200; 711–095-152, Jackson Laboratories) at 37 °C for 45 min. Sections were soaked for 10 s in Hoechst solution H 33342 (1:1000, B2261, Sigma-Aldrich) and were washed once with PBS before mounting with antifading Fluoromount G medium (FP-483331, Interchim). Slides were stored at 4 °C protected from light until picture acquisition.

### Image acquisition and quantification

**Fig. 1:**
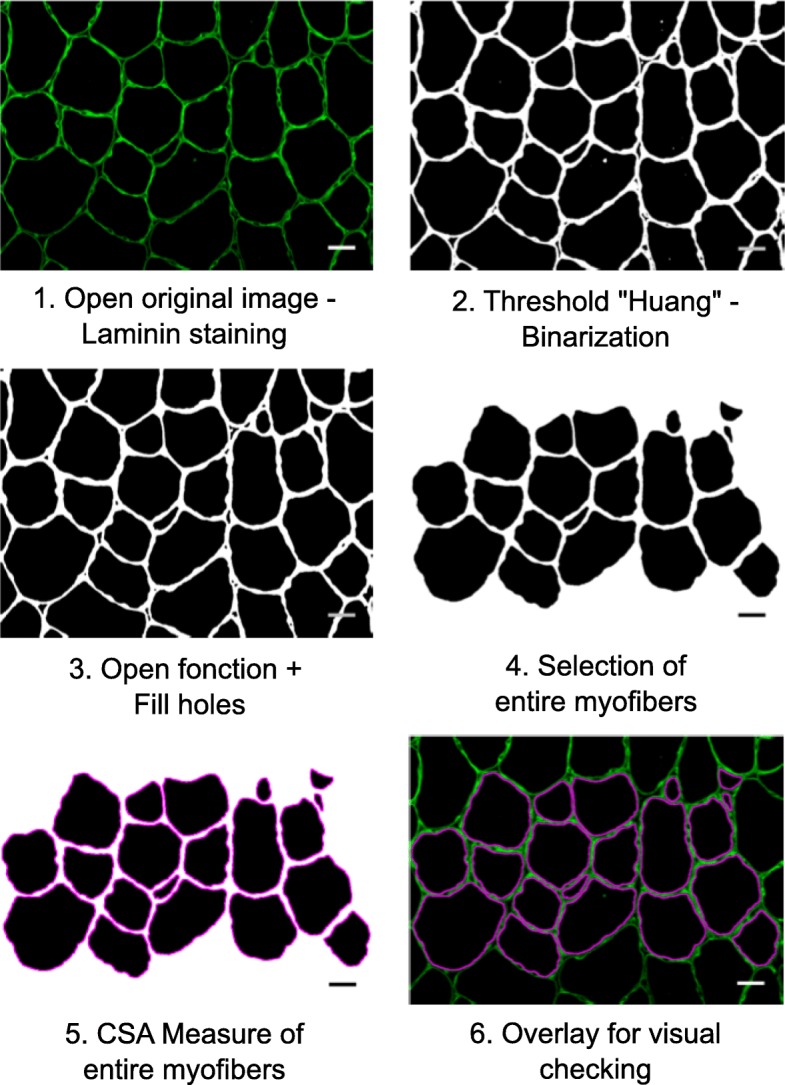
Open-CSAM workflow. Step *1:* When the macro starts, a window automatically opens to select the image to be analyzed (here muscle cryosections immunostained for laminin). Step *2:* Open-CSAM applies the ImageJ threshold “Huang” on the image. Huang threshold was chosen by empiric assays. Threshold application allows image binarization. Step *3*: open function allows to adjust the myofiber contours. Myofibers are filled by the function “fill holes.” Step *4:* Only the entire myofibers are selected to be analyzed. Other selected parameters as circularity and the size are used to avoid the inclusion of too many false myofibers. Step *5:* The area of the selected myofibers is measured. Step *6:* At the end of the measurement, all the region of interests (ROI, here the myofibers) are automatically superimposed for visual checking. It is then possible to manually delete or add new myofibers. Bars = 25 μm

As much as possible, the various conditions to be compared (e.g., WT vs. KO, normal vs. dystrophic muscle) should be recorded in similar conditions (microscope, magnification, exposure time, binning). For Open-CSAM validation and comparison with other softwares, at least 10 images were acquired manually at × 20 of magnification on an Axio Imager.Z1 microscope (Zeiss) connected to a CoolSNAP MYO CCD camera (photometrics) using MetaMorph Software (molecular devices). For whole cryosection analysis, slides were automatically scanned at × 10 of magnification using an Axio Observer.Z1 (Zeiss) connected to a CoolSNAP HQ2 CCD Camera (photometrics). The image of the whole cryosection was automatically reconstituted in MetaMorph Software. Open-CSAM was able to analyze images up to 110 Mo in 8 bit or 220 Mo in 16 bit. In case the final picture was too big to be analyzed at once because of limitation of ImageJ capacities, the picture was split into several parts (two to four) for the automated analysis. Between 2000 and 4000 fibers were analyzed in a few seconds. Open-CSAM workflow is presented in Fig. 1. The detailed macro code and explanations of the various functions are given in Additional file 1: Figure S1. If necessary, at the end of the automated measurement, manual correction is performed using ImageJ. The selection tools in the ROI Manager were used to remove “false” myofibers created by the automation, and the “Freehand selections” tool was used for hand-drawing “lacking” myofibers missed by the automation. These tools were used for full manual quantification. A tutorial is provided in Additional file 2: Figure S2.

MyoVision and MuscleJ analyses were performed as described [2, 8]. SMASH analysis was implemented with a segmentation filter set to 11 [7]. This was empirically determined by testing values between 5 and 12 and visually inspecting the results. For MuscleAnalyzer analysis, the TIFF pipeline was tested following recommendations provided in the tutorial video [11]. Several threshold values were tested from 1 to 0.955 with no difference on the results. In all analyses, the applied size filter was the same as the one used for Open-CSAM (Table 1).

**Table 1:** Recommended size and circularity thresholds

Muscle type	Size threshold	Circularity threshold
D0 (young and old)	200 μm^2*^	0.4
D8	50 μm^2^ then adjust^*^	0.4
D14	100 μm^2^ then adjust^*^	0.4
D28 (young and old)	150 μm^2^ then adjust^*^	0.4
Fib-mdx	50 μm^2^	0.4

^*^If too many small myofibers are missed, progressively decrease the size threshold by testing on 2–3 representative pictures

### Statistical analyses

All images were acquired from at least two independent TA muscles per experimental condition and at least five randomly selected images per muscle were analyzed. The Student *t* test, two-way ANOVA, or Spearman correlation test were used for statistical analyses. *P* < 0.05 was considered significant.

## Results

### Open-CSAM is more accurate than previously described softwares

**Fig. 2:**
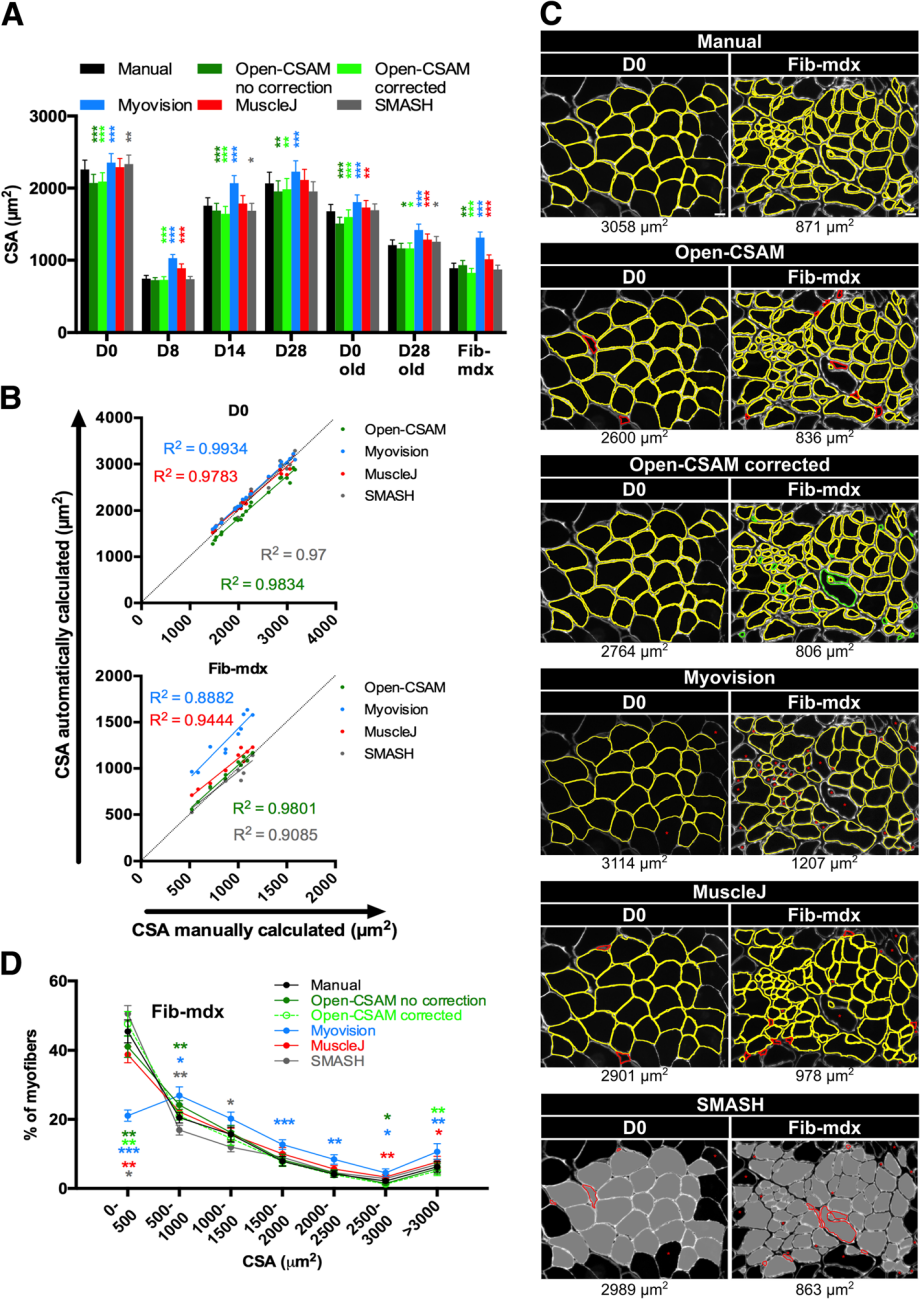
Open-CSAM comparison with MyoVision, MuscleJ, and SMASH softwares. The same pictures were analyzed either by manual measurement or using Open-CSAM (with or without manual correction), MyoVision, MuscleJ, or SMASH softwares. **a** Mean cross-section area (CSA) obtained on various *Tibialis Anterior* (TA) muscles. Muscles were isolated from 8- to 12-week-old mice uninjured (D0) or 8 days (D8), 14 days (D14), and 28 days (D28) post-cardiotoxin (CTX) injury, from 2-year-old mice uninjured (D0 old) or 28 days post-CTX injury (D28 old) and from dystrophic fibrotic mice (Fib-mdx). Results are mean ± SEM of 10 images from 2 muscles (Fib-mdx), 20 images from 2 muscles (D0 and D0 old), 30 images from 3 muscles (D28 and D28 old), 40 images from 4 muscles (D8), and 45 images from 4 muscles (D14). **b** Correlation between manual measurement (*X* axis) and Open-CSAM (without manual correction), MyoVision, MuscleJ, or SMASH (*Y* axis) measurements performed on the same images used in **a**. Each dot represents a picture. The dotted line represents the identity line. **c** Representative images measured manually, by Open-CSAM (before and after correction), MyoVision, MuscleJ, or SMASH softwares. Red fibers were false myofibers identified by the softwares, and green fibers were myofibers not considered by the Open-CSAM software and manually drawn. Lacking myofibers using MyoVision, MuscleJ, or SMASH softwares are shown by red asterisks. **d** Distribution of the CSA obtained with the six methods using the Fib-mdx samples used in **a**. Results are mean ± SEM of 10 images from 2 muscles (Fib-mdx). White bar = 25 μm. *p < 0.05, **p < 0.01, and ***p < 0.001 as compared with manual quantification

In order to test the accuracy of Open-CSAM, its performance was compared with a fully manual quantification, a semi-automated software (SMASH), as well as two fully automated softwares: MyoVision and MuscleJ (Fig. 2). We analyzed TA muscle from various conditions, including uninjured muscle, regenerating muscle at several time points after acute injury (D8 and D14) in young and old mice, and a model of fibrotic dystrophy (Fib-mdx). Quantification was performed on cryosections immunofluorescently labeled with anti-laminin antibody, which labels the myofiber basal lamina. Immunolabeling against dystrophin or sarcolemal proteins was not used as this precludes the analysis in dystrophies where these proteins are lacking or altered. As previously described [2], MyoVision produced significantly higher mean CSA values as compared with manual quantification (Fig. 2a, between 4.3 and 47.7% increase depending on the experimental condition). Mean CSA values obtained with MuscleJ were similar to the manual quantification for uninjured young as well as 14 and 28 days post-injury muscles. However, it gave higher mean CSA values in 8 days post-injury regenerating muscles, old muscles (uninjured and 28 days post-injury), and dystrophic muscles (Fig. 2a, between 3.2 and 19.4% increase). Mean CSA values obtained with SMASH were very close to the manual quantification in uninjured old and young 8- and 28-days post-injury muscles. However, SMASH produced higher mean CSA values in uninjured muscles (young and old) (+ 3 and + 1%, respectively), and lower mean CSA values in 14 days post-injury muscles (− 4%) (Fig. 2a). On the other hand, Open-CSAM without manual correction gave mean CSA values close to those obtained manually, with a slight underestimation (between 2 and 7.3% decrease as compared with manual quantification), except in Fib-mdx muscles where it was slightly overestimated (+ 4.4%) (Fig. 2a). This was explained by the fact that Open-CSAM measured the area inside the basal lamina staining, which corresponds to the true area of the myofiber. With manual quantification, the experimenter tends to draw the limits of the myofibers more on the laminin staining, thus including a small part of the basal lamina, therefore slightly overestimating the myofiber area (Fig. 2a). In the case of Fib-mdx muscle, the overestimation by Open-CSAM was explained by an oversight of small myofibers (see below). During the revision process of this manuscript, MuscleAnalyzer, a customized pipeline within the CellProfiler program allowing fully automated CSA measurement, was released [11]. We tested this pipeline on the same samples as above (Additional file 3: Figure S3). As it strongly overestimated mean CSA as compared with manual quantification (between 38.4% and 254.8% increase depending on the condition), this software was not considered for further analysis.

Despite an increased CSA value obtained using MyoVision, the correlation between MyoVision and manual quantification for each picture was strong in uninjured muscles (young and old mice), as well as 14 and 28 days post-injury regenerating muscles (Fig. 2b and Additional file 4: Figure S4A, *R*^2^ > 0.95), suggesting that in these conditions, CSA overestimation by MyoVision was similar on all the pictures and did not introduce a specific bias. On the contrary, the correlation was much lower on muscles at 8 days post-injury, 28 days post-injury in old mice and in fibrotic muscles (Fig. 2b and Additional file 4: Figure S4A, R^2^ < 0.89), which represent conditions exhibiting smaller myofibers and/or defects in regeneration. Similarly, overall correlation between MuscleJ and manual quantification was very strong in uninjured muscles (young and old mice, Fig. 2b and Additional file 4: Figure S4A, R^2^ > 0.97). Although this correlation was lower on young 8 days and old 28 days post-injury as well as on fibrotic muscles, it was better than MyoVision. Finally, correlation was lower with MuscleJ than with MyoVision for the 14 and 28 days post-injury conditions. Correlation between SMASH and manual quantification was strong in uninjured young muscles (Fig. 2b, *R*^2^ = 0.97). However, correlation was less good when compared with MyoVision and MuscleJ in 28 days post-injury muscles (young and old mice, Additional file 4: Figure S4A, *R*^2^ = 0.8352 for young and 0.9278 for old). For all other conditions, SMASH accuracy compared with manual quantification was intermediate between that of MyoVision and of MuscleJ (Additional file 4: Figure S4A, 0.8489 < *R*^2^ < 0.9364). These data indicate that the differences observed between manual quantification and MyoVision, MuscleJ, or SMASH are condition-dependent, thus introducing a bias. We then used Open-CSAM, first without applying any manual correction. The correlation between Open-CSAM and manual quantification was better or equivalent to that of MyoVision, MuscleJ, or SMASH in all the analyzed experimental conditions (Fig. 2b and Additional file 4: Figure S4A, *R*^2^ > 0.9454). This suggests that Open-CSAM performance was more consistent through the various experimental conditions.

### Manual correction is necessary for best accuracy

Figure 2c shows two examples of quantification performed on a single image from an uninjured muscle (left panel) and a dystrophic fibrotic muscle (right panel). In the uninjured muscle picture, Open-CSAM and MuscleJ identified two false myofibers (red lines), which were manually removed with Open-CSAM (Open-CSAM corrected). On the other hand, MyoVision missed two myofibers (red asterisks) while SMASH both identified three false myofibers and missed two myofibers. However, as the myofiber size was quite homogeneous in this picture, this did not really impact the calculated CSA value. In the fibrotic muscle picture (Fig. 2c, right panel), Open-CSAM identified 7 false myofibers (red lines) and missed 16 myofibers that were manually corrected afterwards (green lines). MyoVision missed 33 myofibers (red asterisks) and identified 1 false fiber, which could be excluded by size filter in MyoVision software (red line), inducing a 29% artificial increase in CSA, as compared with manual quantification. Similarly, MuscleJ identified 7 false myofibers (red lines) and missed 14 fibers (red asterisks), leading to a 12.3% increase as compared with manual quantification. Finally, SMASH missed 14 myofibers and misidentified 9 fibers, leading to an 8.2% decrease of mean CSA compared to manual quantification. The distribution of the myofiber CSA in Fib-mdx muscles (same samples as in Fig. 2a) clearly showed that MyoVision and MuscleJ, and to a lower extent, Open-CSAM, preferentially missed the small myofibers (< 500 μm^2^) (Fig. 2d), thus artificially overestimating the mean CSA. On the contrary, SMASH overestimated the proportion of small myofibers. Taken together, these results show that Open-CSAM accuracy is more consistent as compared with other softwares through various experimental conditions. However, despite its performance, manual correction is necessary for best accuracy. Open-CSAM was designed to enable easy manual correction, allowing the user to directly draw missing fibers and delete the false ones at the same time in ImageJ (Additional file 2: Figure S2). As shown in Additional file 4: Figure S4B, manual correction applied to Open-CSAM improved the measurement accuracy in all conditions (*R*^2^ > 0.97) except in old mice 28 days post-injury (*R*^2^ = 0.9665 before and 0.962 after correction). In Fib-mdx muscle, this was associated with a convergence of the overall myofiber size distribution towards the distribution obtained with manual quantification (Fig. 2d), notably with a better consideration of small myofibers (< 500 μm^2^).

### Whole section analysis is necessary for best accuracy

**Fig. 3:**
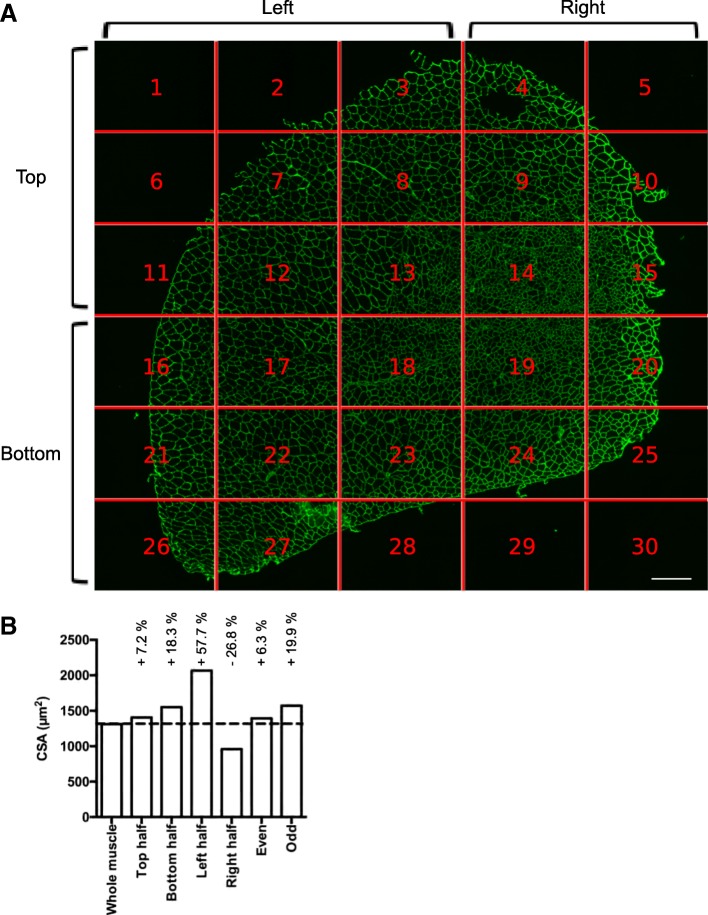
Whole muscle analysis by Open-CSAM. **a** Whole reconstitution of a laminin-stained cryosection of a TA muscle 28 days post-CTX injury (30 pictures were automatically recorded and assembled by MetaMorph software). The position of each individual image is highlighted by the red lines. White bar = 250 μm. **b** Mean cross-section area obtained after various subsettings of pictures in **a**

CSA analysis is usually performed on a subset of images randomly taken throughout the muscle section. Depending on the experimenter, the number of images and thus the number of analyzed myofibers can be variable. Moreover, within the same muscle, myofiber size can be quite heterogeneous and a bias can be introduced depending on the choice of the pictures. Figure 3a shows an example of an entire reconstituted muscle picture. We measured CSA on individual images, calculated the mean CSA on several subsets of images, and compared the results with the CSA obtained on the whole section. When the measurement was made only using a subset of pictures, the myofiber CSA differed from 5 to 28% to that measured on the whole muscle (Fig. 3b). We particularly observed that CSA was overestimated when the left half of the muscle was measured, that corresponded in this example to the peripheral part of the muscle where regeneration is ended. Inversely, measuring the right part of the muscle, here corresponding to smaller regenerating myofibers localized at the center of the muscle, underestimated CSA value. Thus, because TA regeneration after a toxic injury is a centripetal process that ends first at the periphery of the muscle, the whole muscle section should be analyzed when measuring CSA of regenerating muscle in order to obtain an unbiased picture of the process. Moreover, as in diseased muscle, foci of fiber damage or remodeling can occur anywhere, analyzing the whole section also insures an accurate measurement in this context.

### Use of Open-CSAM is robust among users

**Fig. 4:**
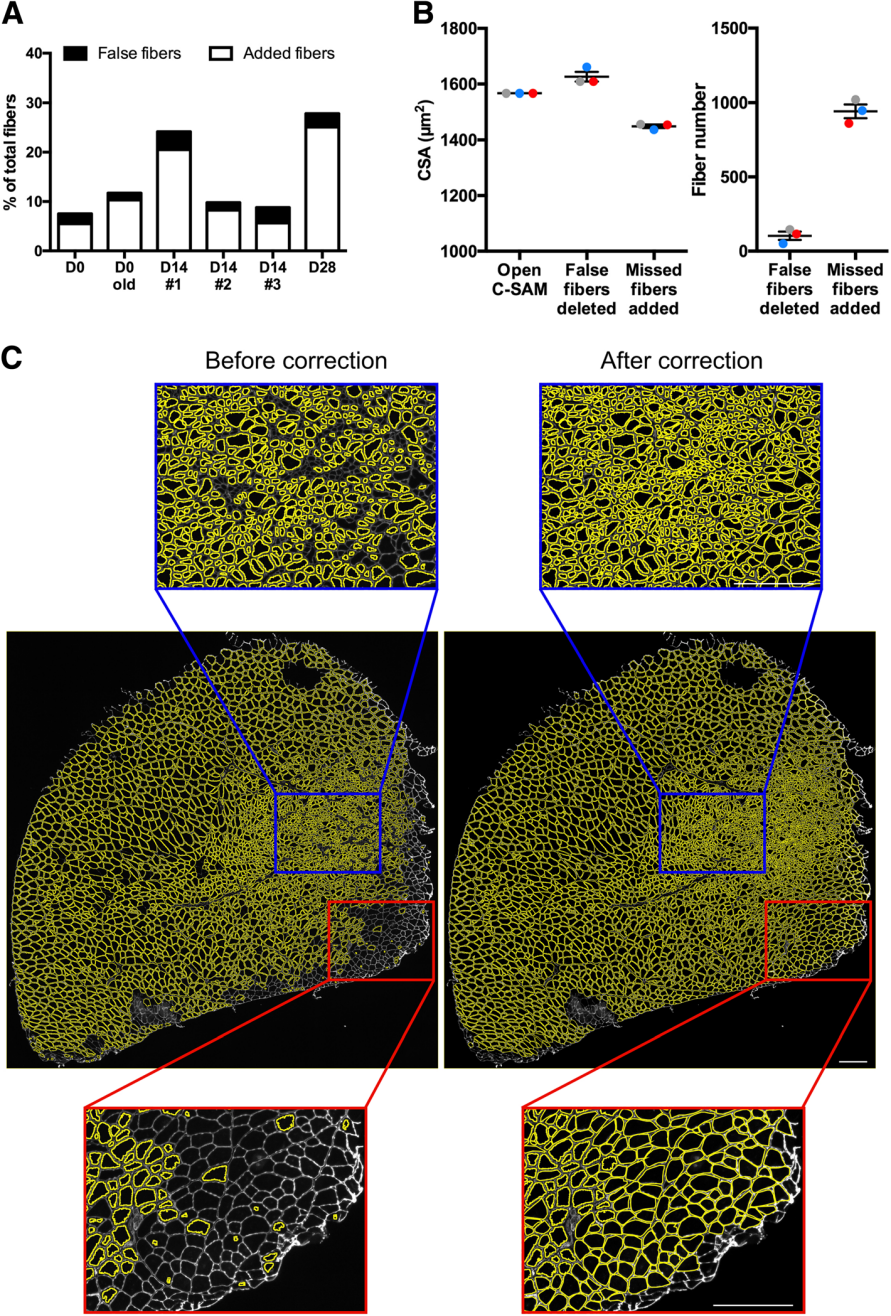
Manual corrections after Open-CSAM analysis. **a** Percentage of false myofibers detected (black histograms) and missed myofibers (white histograms) by Open-CSAM that needed manual correction for TA muscles from young and old uninjured mice as well as from 3 muscles analyzed 14 days post-CTX injury. **b**, **c** Open-CSAM analysis of a TA muscle 28 days post-CTX injury. **b** CSA (left graph) and number of fibers manually corrected (right graph) after analysis by Open-CSAM by three different users (each color represents a single user). **c** Whole image of the TA muscle analyzed in **b** showing myofibers (yellow) detected by Open-CSAM before (left panel) and after (right panel) manual correction. Blue and red boxes represent zoom-in examples of two specific areas that needed extensive manual correction. White bar = 250 μm

In a whole muscle section, the number of manual corrections can be variable depending on the quality of the muscle section and of the immunostaining. Considering a high quality of both, we found that the amount of false plus lacking fibers, which needed to be manually removed or re-drawn, ranged from 5% in non-damaged muscles to 10 to 25% in damaged muscles (Fig. 4a). As the manual corrections after Open-CSAM analysis can represent up to 25% of the myofibers, thus potentially introducing variability among users, we tested the robustness of this method. To do so, three independent experimenters analyzed the same image taken from an adult TA muscle 28 days post-CTX injury, where the majority of the muscle has fully regenerated and some central areas are still regenerating (Fig. 4b, c). This muscle was chosen because of the high number of required manual corrections. Using a 100 μm^2^ size filter and a 0.4 circularity threshold (see Additional file 1: Figure S1 and Table 1), Open-CSAM identified 3757 fibers (Fig. 4c, left images), resulting in a CSA of 1566.8 μm^2^ (Fig. 4b, left). As shown in Fig. 4c, two areas showed a large number of fibers missed by Open-CSAM. The first area (blue box) was composed of fibers showing a high variability in size where Open-CSAM missed mainly very small fibers. The second area (red box) was composed of medium- to large-sized fibers but with a lower laminin staining intensity. In this area, Open-CSAM failed to identify most of the fibers. Depending on the user, 50 to 145 false fibers were deleted and 860 to 1019 fibers were manually added (Fig. 4b, right). Despite this variability in the number of manual corrections performed by each user, the corrected CSA reached 1454, 1437, and 1456 μm^2^, meaning that variability between users was below 1%. Altogether, these results show that despite the manual corrections required by Open-CSAM, there is no bias introduced by the experimenter.

### Open-CSAM is an accurate tool for whole muscle CSA analysis in regenerating muscle

**Fig. 5:**
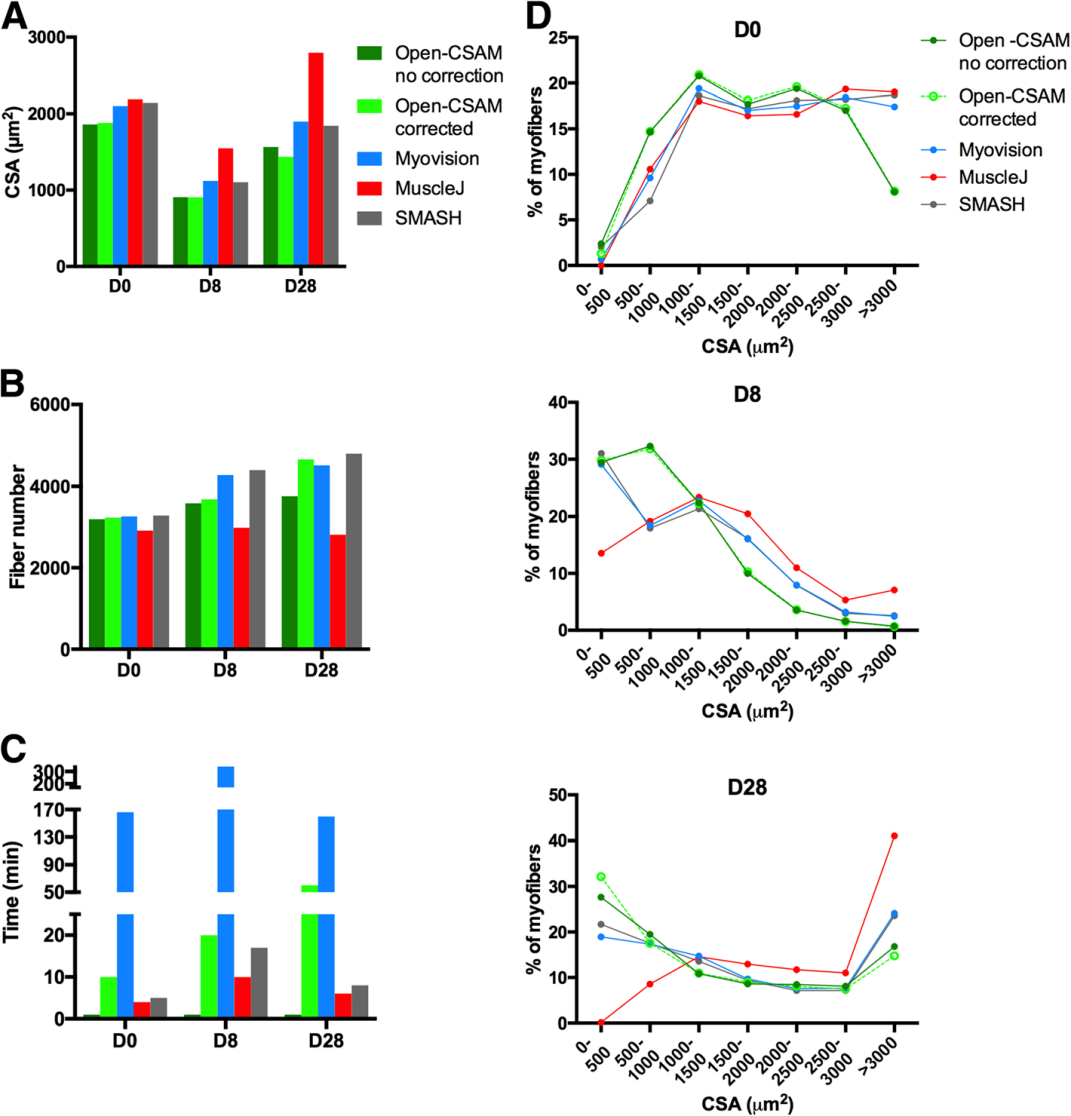
Comparison of Open-CSAM, MyoVision, MuscleJ, and SMASH CSA quantification on whole muscle sections. CSA was measured using Open-CSAM (with or without manual correction), MyoVision, MuscleJ, or SMASH on whole TA muscle images obtained from uninjured (D0) or 8 days (D8) and 28 days (D28) post-CTX injury. **a** Mean CSA measured by the different softwares. **b** Number of fibers identified by the softwares. **c** Total analysis time required by the softwares. **d** Distribution of CSA obtained by the five methods on D0 (top graph), D8 (middle graph), and D28 (bottom graph) whole muscles

As we showed that CSA quantification with Open-CSAM was highly accurate on individual images in various experimental conditions and that whole muscle analysis was necessary, we tested its performance on several whole muscle images and compared it to fully automated quantification by MyoVision and MuscleJ, as well as to SMASH. We analyzed whole muscle images from young uninjured, 8 days post-injury and 28 days post-injury mice (Fig. 5 and Additional file 5: Figure S5). As we showed for × 20 individual pictures in uninjured muscle (Fig. 2), MyoVision, MuscleJ, and SMASH modestly overestimated the CSA as compared with corrected Open-CSAM (Fig. 5a, + 11.7%, + 16.5%, and + 13.9%, respectively) (Fig. 5a). On the same muscle, manual correction had negligible impact on CSA obtained by Open-CSAM, highlighting its high performance on uninjured whole muscle section (Fig. 5a, − 1.1% before correction). Corroborating this observation, CSA distribution was identical for Open-CSAM before and after correction (Fig. 5d). On the contrary, MyoVision, MuscleJ, and SMASH overlooked small fibers (< 1000 μm^2^) and overestimated bigger fibers (> 3000 μm^2^). In the D8 condition, where small fibers represent an important part of the whole muscle section area and the D28 condition, where small myofibers are still concentrated in specific areas (see Fig. 4), MyoVision (+ 24 and + 32.1%, respectively), MuscleJ (+ 71.2 and + 92.3%, respectively), and SMASH (+ 22 and 26.8%, respectively) accounted for a much higher CSA than Open-CSAM. This is mainly due to the non-detection of the small myofibers (Fig. 5d) and, in the case of MuscleJ, to the merging of several myofibers into one (Additional file 5: Figure S5B, Fig. 5b). Again, correction in Open-CSAM induced a negligible change in CSA measurement on day 8 post-injury muscle (Fig. 5a, + 0.3% before correction). Finally, manual correction of Open-CSAM in day 28 post-injury muscle corrected a slight underestimation of small fibers (Fig. 5d), leading to an artificial increase in CSA (Fig. 5a, + 7.8% before correction).

The main defect of Open-CSAM is the time of experimenter that is required for the complete implementation of CSA measurement. Although running Open-CSAM macro in ImageJ took less than 1 min (even for the whole muscle pictures), which was faster compared to other softwares (4 to 10 min for MuscleJ, 5 to 17 min for SMASH, and 166 to 340 min for MyoVision), the post-automation manual corrections lasted between 10 and 60 min in the examples provided (Fig. 5c, Additional file 5: Figure S5). However, those corrections are required for achieving a full analysis of the whole muscle in order to provide an accurate CSA value. Taken together, these results show that Open-CSAM is a highly accurate tool to measure myofiber CSA on whole muscle section images from different experimental conditions, including regenerating muscle.

## Discussion

Histology is the gold standard technique to assess some characteristics of skeletal muscle. Measurement of myofiber CSA is classically used as a parameter of normal and pathological skeletal muscle regeneration after labeling with anti-laminin or anti-dystrophin antibodies. However, there are various strategies to analyze images and CSA measurement that can give highly heterogeneous results. On the one hand, manual quantification, consisting in manually drawing the myofiber contour to measure the area, is extremely time-consuming and potentially variable between experimenters as it is meticulousness-dependent. According to our experience, it takes about 1 h to draw 1000 fibers. On the other hand, automated programs that were developed to reduce the impact of the experimenter, and to save time, give satisfactory results in uninjured muscle [2–8] but fail to be accurate in regenerating muscle.

Among the recent described softwares are SMASH, MyoVision, and MuscleJ. SMASH is a MATLAB application which is also available as a free Windows program (SMASH Stand Alone). MyoVision is a program operating in Windows environment whereas MuscleJ is a macro working in ImageJ program (Table 2). They were developed to automate, with various levels of flexibility, the quantification of several muscle parameters including fiber number, CSA, myonuclei number and fiber type distribution. As previously described [2], MyoVision showed a significant CSA overestimation as compared with manual quantification. This could be explained by the stronger overlapping of MyoVision outlines with the laminin staining (Fig. 2c), thus integrating a part of the basal lamina into the myofiber area. Despite this overestimation of the mean CSA as compared with manual quantification, MyoVision performed accurately on isolated images from uninjured muscles. However, regenerating, old and dystrophic muscles are characterized by high myofiber size heterogeneity. In this context, MyoVision measurement was still good at day 14 and 28 after injury, i.e. at time points when regenerating myofibers are already formed. On the contrary, correlation with manual estimation was much lower in muscles at 8 days post-injury in young mice, at 28 days post-injury in old mice and in muscles of dystrophic fibrotic mice. These three conditions are characterized by a lower mean CSA and the presence of high numbers of very small myofibers, which are not considered by MyoVision, thus overestimating the mean CSA. MuscleJ software was efficient in measuring mean CSA in individual × 20 pictures from uninjured young as well as 14 and 28 days post-injury muscles. However, it measured higher mean CSA values in 8 days post-injury regenerating muscles, old muscles (uninjured and 28 days post-injury) and dystrophic muscles, even though the values were closer to manual quantification than that calculated by MyoVision. Here again, this was due to an oversight of small myofibers. Finally, SMASH showed high accuracy in uninjured young muscles. For the other conditions, even though mean CSA was relatively close to manual quantification, the accuracy was very variable between images, leading to overestimation of small myofibers in Fib-mdx (Fig. 2d) and oversight of these same small fibers in regenerating muscles (Fig. 5d), making it not reliable to analyze and compare various conditions.

**Table 2:** Comparison of software characteristics

Program	Software base	OS	Minimal configuration tested		Manual correction
			PC	MAC	
Open-CSAM	ImageJ plugin	Mac OS/Windows	Windows 10-Intel core i3 CPU 550 3.2 GHz-4 Go RAM	Mac OS 10.12/Intel core i3–3.2 GHz/12 Go RAM	Yes—during the analysis ➔ Draw/delete ImageJ tools
MyoVision	Windows application	Windows	Windows 10-Intel core i3 CPU 550 3.2 GHz-4 Go RAM (single image analysis)/Windows 7-Intel core i5 vpro 3.1 GHz/16 Go RAM (whole muscle analysis)	Not applicable	No
MuscleJ	ImageJ plugin	Mac OS/Windows	Windows 10-Intel core i3 CPU 550 3.2 GHz-4 Go RAM	Mac OS 10.13/Intel core i5–2.7 GHz/16 Go RAM	Yes—after the analysis ➔ Open picture ➔ Import ROI file on the picture ➔ Draw/delete ImageJ tools
SMASH Stand Alone	Windows application	Windows	Windows 10-Intel core i3 CPU 550 3.2 GHz-4 Go RAM	Not applicable	Yes—during the analysis ➔ Step 1 draw—validate ➔ Step 2 delete—validate (cumbersome: small screen, no back and forth allowed between steps 1 and 2)

In order to provide a tool usable in various biological conditions, we developed Open-CSAM, an ImageJ macro allowing the automatic measurement of CSA with the possibility for the experimenter to apply manual corrections afterwards. CSA values obtained with Open-CSAM were very close to the values obtained manually in all tested conditions. Generally, Open-CSAM omitted only few myofibers in muscles containing small fibers (regenerating muscle, old muscle, dystrophic muscles). Then, lacking myofibers are easily manually drawn and incorporated into the analysis using ImageJ, which is not possible using the fully automated softwares. The main drawback of Open-CSAM is that it identifies some false myofibers in the interstitial space between myofibers, notably when this space is large in early regenerating and fibrotic muscles. This issue can be overcome by increasing the size and the circularity thresholds but this also increases the risk of omitting real myofibers. Because it is faster to delete false myofibers than manually drawing lacking myofibers, we recommend to keep thresholds as low as possible to reduce the number of lacking myofibers. We therefore provided starting thresholds for the different conditions we have tested, that have to be adjusted depending on the specific experiment (Table 1). According to our experience, the false fibers identified by Open-CSAM represented less than 5% of the total fibers in all the muscles we have analyzed so far.

CSA measurement is usually performed on a subset of images randomly taken throughout the muscle section. However, myofiber size is quite heterogeneous within the same muscle, and we showed that considering only a part of the muscle section led to the introduction of bias depending on the image selection, potentially hiding a relevant phenotype or artificially creating a non-relevant one. Of note, image acquisition and reconstitution of the whole muscle section is automatically performed, saving time, as compared with manually recording random pictures. Then, Open-CSAM is capable of measuring the myofiber CSA on the whole section in a few seconds. Depending on the size of the image, it may be necessary to split it in two or four parts. This may be also useful when the laminin labeling intensity is not homogenous on the entire section since Open-CSAM may omit faint labeled areas.

When measuring CSA on whole muscle section pictures, using uninjured or 8-day and 28-day regenerating muscles, we found that Open-CSAM (including the manual post-automation corrections) was the most accurate way to measure myofiber CSA. MuscleJ program was efficient at implementing CSA from uninjured muscle (still with overestimated values) but failed to accurately measure myofiber CSA of regenerating muscle, due to failure to detect small fibers and to merging of several myofibers into one. Even though MuscleJ was built as a ready-to-use toolbox to avoid experimenter intervention, post-automation corrections are possible. Indeed, the user may generate a file containing the ROIs analyzed that can be imported back into ImageJ to perform manual corrections in a similar way as Open-CSAM. However, given its poor performance on whole regenerating muscle section as compared with Open-CSAM, the amount of manual corrections and thus the time spent would be not competitive towards Open-CSAM measurement. SMASH and MyoVision gave similar results, as they moderately overestimated mean CSA in uninjured muscle, an overestimation that was amplified in 8-day and 28-day regenerating muscles which contain a higher proportion of small rounded-shape myofibers. MyoVision was very slow in implementing the analysis of the whole muscle images (between 166 and 340 min), making it not competitive towards manual measurement. Moreover, it does not allow manual correction. SMASH was faster (between 5 and 17 min), and it was designed to allow the user to perform manual corrections during the analysis. However, the correction process has several drawbacks. First, the application cannot be displayed into full screen, which makes inconvenient to easily detect the myofiber delimitations. Second, after the segmentation, the correction process is composed of 2 sequential steps. The first step allows to manually split merged fibers or draw the missed ones. After validation of the drawing, the second step allows manual deletion of the false fibers identified by the program. Unfortunately, at this point, it is not possible anymore to come back to correct merged or missed fibers that would have appeared after the second step. Given this lack of flexibility, performing manual correction in Open-CSAM is much easier and faster.

According to our experience with Open-CSAM, the amount of manual corrections required after the automatic measurement depends on the status of the muscle, the quality of the cryosection, and the quality of the immunolabeling. We recommend performing a new cryosection and/or labeling rather than trying to analyze poor quality images. Typically, if we consider a high-quality image, the analysis of an uninjured TA lasts less than 15 min to process 2000 to 3000 myofibers. In the worst condition we experienced, processing of a regenerating TA can last from 15 min to 1 h for the experimenter to obtain an accurate measurement of myofiber CSA (as compared with 1 h per 1000 fibers in the case of a full manual quantification, thus 2–3 h per muscle). Moreover, we showed that despite the relatively high level of manual correction required for some muscles, the difference in corrected CSA obtained by independent experimenters was negligible (less than 1%), highlighting the robustness of this method.

## Conclusion

The use of Open-CSAM program on whole muscle sections is a powerful strategy to measure myofiber CSA of muscles from various experimental conditions in an easy, highly accurate, and reproducible way, providing values very close to the absolute values. This user-friendly (tutorial in Additional file 2: Figure S2) method is semi-automated and therefore requires the commitment of the experimenter (who is still the best expert to define what a myofiber is), allowing the most accurate CSA measurement of regenerating muscle so far.

## Additional files

Additional file 1: **Figure S1.** Macro to be run for the implementation of Open-CSAM program (left) and related explanations of the functions (right column). (PDF 31.1 kb)

Additional file 2: **Figure S2. **Tutorial for the use of Open-CSAM. (PDF 785 kb)

Additional file 3: **Figure S3.** The same pictures as in Fig. 2 were analyzed either by manual measurement or using MuscleAnalyzer. The mean CSA obtained with the two methods is shown. ***p < 0.001 as compared with manual quantification by two-way ANOVA analysis. (PDF 80 kb)

Additional file 4: **Figure S4.** Mean CSA was measured manually and with Open-CSAM, MyoVision, MuscleJ, or SMASH softwares on the same samples as described in Fig. 2a. Muscles were isolated from 8- to 12-week-old mice 8 days (D8), 14 days (D14), and 28 days (D28) post-CTX injury, from uninjured (D0 old) or 28 days post-CTX injury (D28 old) 2-year-old mice. **A** The correlation between manual measurement (*X* axis) and Open-CSAM (without manual correction), MyoVision, MuscleJ, or SMASH (*Y* axis) measurements is presented. **B** Correlation between manual measurement (*X* axis) and Open-CSAM (*Y* axis) before and after manual correction. Each dot represents a picture. The dotted line represents the identity line. (PDF 833 kb)

Additional file 5: **Figure S5.** CSA was measured using Open-CSAM, MyoVision, MuscleJ, or SMASH softwares on whole TA muscle images obtained from uninjured (D0) or 8 days (D8) and 28 days (D28) post-CTX injury. **A** Pictures showing the myofibers (yellow shapes except for SMASH which is in gray) detected by Open-CSAM, MyoVision, SMASH, and MuscleJ. The white dotted lines show where the images were split for Open-CSAM and MyoVision analysis. **B** Red boxes represent zoom-in examples of specific areas obtained by MuscleJ. Red asterisks show examples of group of myofibers that are merged. White bar = 250 μm. (PDF 12792 kb)

## Abbreviations

CSA: Cross-sectional area
CTX: Cardiotoxin
Fib-mdx: Fibrotic dystrophic mice
ROI: Region of interest
TA: Tibialis anterior
