# Supplementary material for: Open-CSAM, a new tool for semi-automated analysis of myofiber cross-sectional area in regenerating adult skeletal muscle
Source: Skelet Muscle. 2019 Jan 8;9:2. doi: 10.1186/s13395-018-0186-6 (PMC6323738; doi:10.1186/s13395-018-0186-6)
Supplement: Supplementary file 1 — Figure S1. Macro to be run for the implementation of Open-CSAM program (left) and related explanations of the functions (right column). (PDF 31.1 kb) [file 13395_2018_186_MOESM1_ESM.pdf]

Functions	Explanations
// Initialisation<br>roiManager("Reset");<br>setBackgroundColor(0, 0, 0);<br>run("Close All");<br><br>if (isOpen("Summary")) {<br>selectWindow("Summary");<br>run("Close");<br>}<br>if (isOpen("Results")) {<br>selectWindow("Results");<br>run("Close");<br>}	This function is used for rebooting all the tools used for the measure of myofiber size and for closing all the opened windows (image and others) to avoid interference with the macro during the analysis of a new image.
// Open muscle section image<br>open();<br>run("Set Scale...", "distance=1 known=**0.645** unit=µm");<br>original = getImageID();<br>run("Duplicate...", " ");<br>mask = getImageID();	This function allows a new image to open. It also allows to scale the picture. So it is very important to know the pixel camera and to adjust it depending on the microscope-camera used in the following section in bold:<br>run("Set Scale...", "distance=1 known=**0.645** unit=µm");
setAutoThreshold("Huang");	We use here the threshold filter “Huang”. By empiric measures, it appeared to be the most appropriate threshold filter to convert images in a 8-bit binary image that is required for the next steps.
setOption("BlackBackground", false);<br>run("Convert to Mask");<br>run("Options...", "iterations=2 count=1 do=Open");<br>run("Fill Holes");	These functions allow to close the mask of the myofibers which are not completely closed after the threshold and to eliminate all isolated particles within the myofiber mask.
run("Set Measurements...", "area shape feret's area_fraction display add redirect=None decimal=3");<br>run("Analyze Particles...", "size=100-Infinity circularity=0.4-1.00 show=Masks display exclude summarize add in_situ");	These functions allow to define the analyzed parameters (area is the most important) and the characteristics of particles to be analyzed (size and circularity). The size of the particles must be adjusted depending on the characteristics of the sample (*e.g.* smaller myofibers are observed at early stages of regeneration than at late stages). Circularity (4π x Area / Perimeter^2) defines if the object is a perfect circle (circ=1) or a very elongated polygon (circ close to 0). Circularity is used to exclude the most elongated and polygonal objects (such as very elongated myofibers and non myofiber objects). It also allows to exclude oblique sections. We consider only sections with a mean circularity >0.6.
roiManager("Show All");<br>selectImage(original);<br>run("Enhance Contrast", "saturated=0.35");<br>roiManager("Show All");	These parameters allow to show all the particles measured in the original image. This function is very useful to check if the measure is accurate, and to do some corrections if necessary (delete fake myofibers and draw the lacking myofibers with the “freehand selections” tool).
