# Supplementary material for: Open-CSAM, a new tool for semi-automated analysis of myofiber cross-sectional area in regenerating adult skeletal muscle
Source: Skelet Muscle. 2019 Jan 8;9:2. doi: 10.1186/s13395-018-0186-6 (PMC6323738; doi:10.1186/s13395-018-0186-6)
Supplement: Supplementary file 2 — Figure S2. Tutorial for the use of Open-CSAM. (PDF 785 kb) [file 13395_2018_186_MOESM2_ESM.pdf]

Tutorial

Open-CSAM works with ImageJ or Fiji software.

1- Drag the Open-CSAM macro on ImageJ window to open it (Open-CSAM.ijm downloaded from Image J website), or create the macro (copy paste the macro program (in red in Supplemental Figure 1) in the macro-record function of ImageJ).
2- Adjust the pixel size depending on the pixel/camera of the microscope used to record pictures: *run("Set Scale...", "distance=1 known=**0.645** unit=µm");*
(in the above example, 0.645 in bold is the pixel camera)
Open-CSAM uses a size and a circularity filter to exclude extra-cellular spaces from the analysis. Depending on the experimental condition, it can be relevant to adjust the size and the circularity of the selected myofibers that have to be analyzed in the following section of the program in bold:
run("Analyze Particles...", "size=**100**-Infinity circularity=**0.4**-1.00 show=Masks display exclude summarize add in_situ");
3- Run Open-CSAM (*Macros* ➔ *Run macro*).
4- A window automatically opens to choose the picture to be analyzed (Open-CSAM can read several formats: TIFF, JPEG, PNG but we preferentially use TIFF format).
5- Let Open-CSAM analyzing the picture.
6- At the end of the analysis, myofiber shapes analyzed by Open-CSAM are shown in the original picture for a visual check of the analysis.
If the analysis is perfect, results can be exported into excel.

If corrections are required:
a- deleting false fibers: select and delete the false fibers directly on the picture (example in blue in the picture), they will be deleted from the ROI Manager.
b- drawing missing fibers: with the freehand tool, draw missing fibers. When you have finished to draw one fiber, press Command+T (Mac.OS) or CTL+T (Windows) to add the new myofiber to the ROI manager.

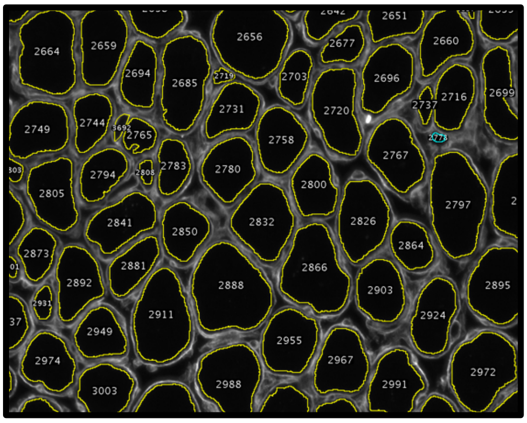

7- When all the corrections are made, close the previous *Results* window and in the ROI manager, select all the ROIs and click on *Measure* to measure the area of all the fibers/ROI. Results (see a screenshot below) can be exported into excel.

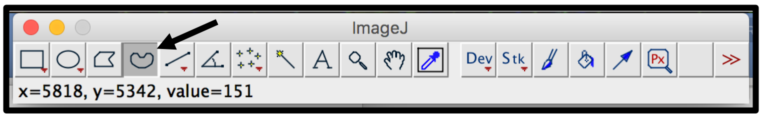

Results

	Label	Area	Circ.	Feret	%Area	FeretX	FeretY	FeretAngle	MinFeret
1	A16-79 TAD.tif	2792.360	0.596	82.343	100	2248.470	81.270	95.845	48.627
2	A16-79 TAD.tif	2303.946	0.451	78.300	100	2503.245	131.580	11.402	44.272
3	A16-79 TAD.tif	1027.998	0.556	52.002	100	2660.625	118.035	136.005	30.407
4	A16-79 TAD.tif	1735.240	0.457	80.003	100	2636.115	118.680	119.458	35.962
5	A16-79 TAD.tif	1985.271	0.641	65.403	100	2465.190	185.760	84.908	38.329
6	A16-79 TAD.tif	2236.134	0.452	74.161	100	2712.225	133.515	139.939	47.792
7	A16-79 TAD.tif	2694.594	0.663	71.830	100	2254.920	198.015	4.635	54.807
8	A16-79 TAD.tif	2143.777	0.649	65.078	100	2342.640	207.045	50.631	46.751
9	A16-79 TAD.tif	4374.919	0.683	97.256	100	2500.665	157.380	111.801	59.757
10	A16-79 TAD.tif	1374.131	0.571	63.358	100	2792.205	161.250	144.530	35.572
