# Supplementary figures and images for: Open-CSAM, a new tool for semi-automated analysis of myofiber cross-sectional area in regenerating adult skeletal muscle
Source: Skelet Muscle. 2019 Jan 8;9:2. doi: 10.1186/s13395-018-0186-6 (PMC6323738; doi:10.1186/s13395-018-0186-6)

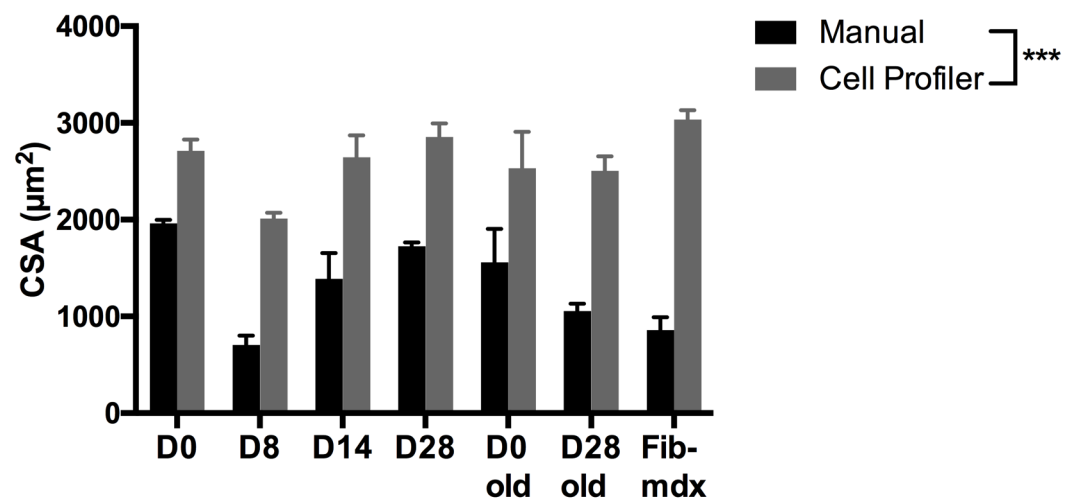

Figure S3

Supplement: Supplementary file 3 — Figure S3. The same pictures as in Fig. 2 were analyzed either by manual measurement or using MuscleAnalyzer. The mean CSA obtained with the two methods is shown. ***p < 0.001 as compared with manual quantification by two-way ANOVA analysis. (PDF 80 kb) [file 13395_2018_186_MOESM3_ESM.pdf]

**A**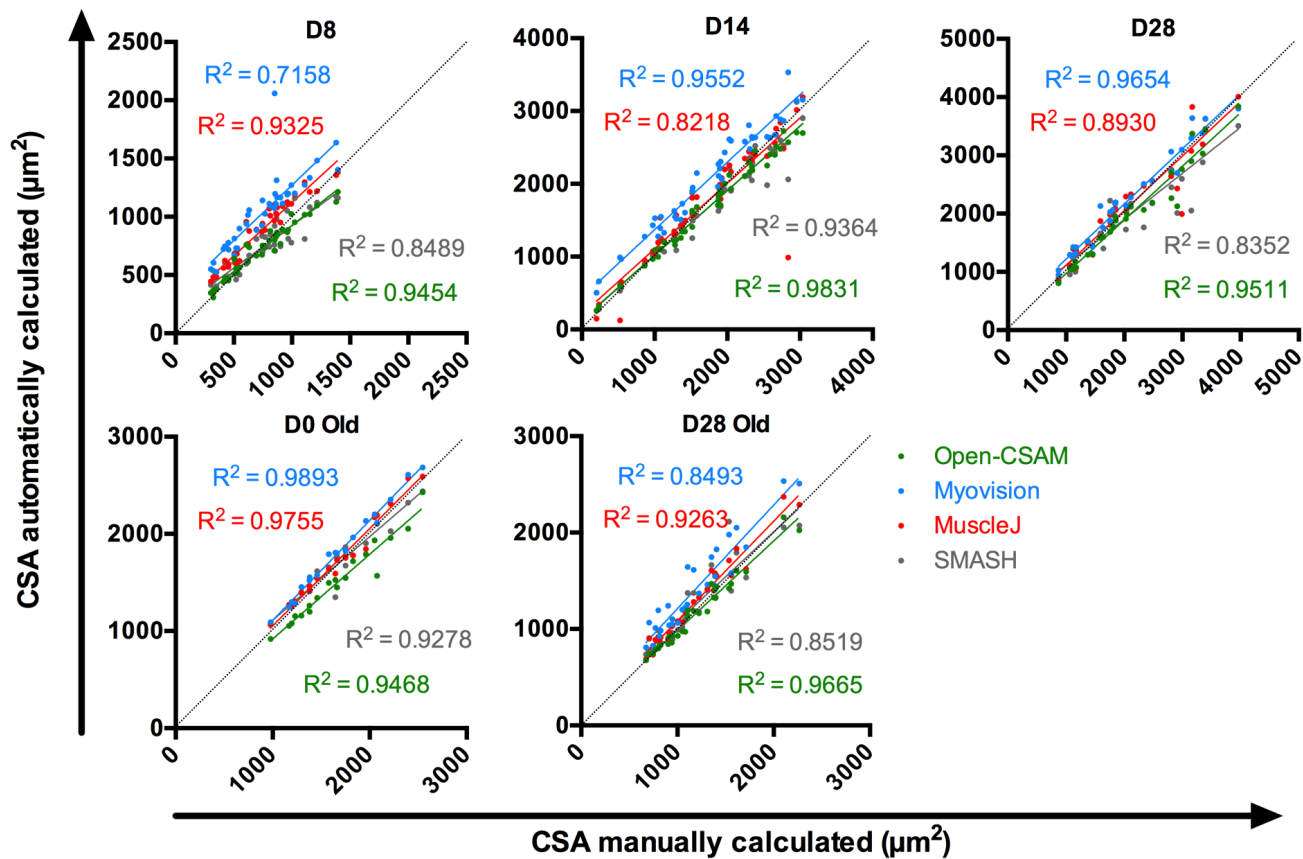**B**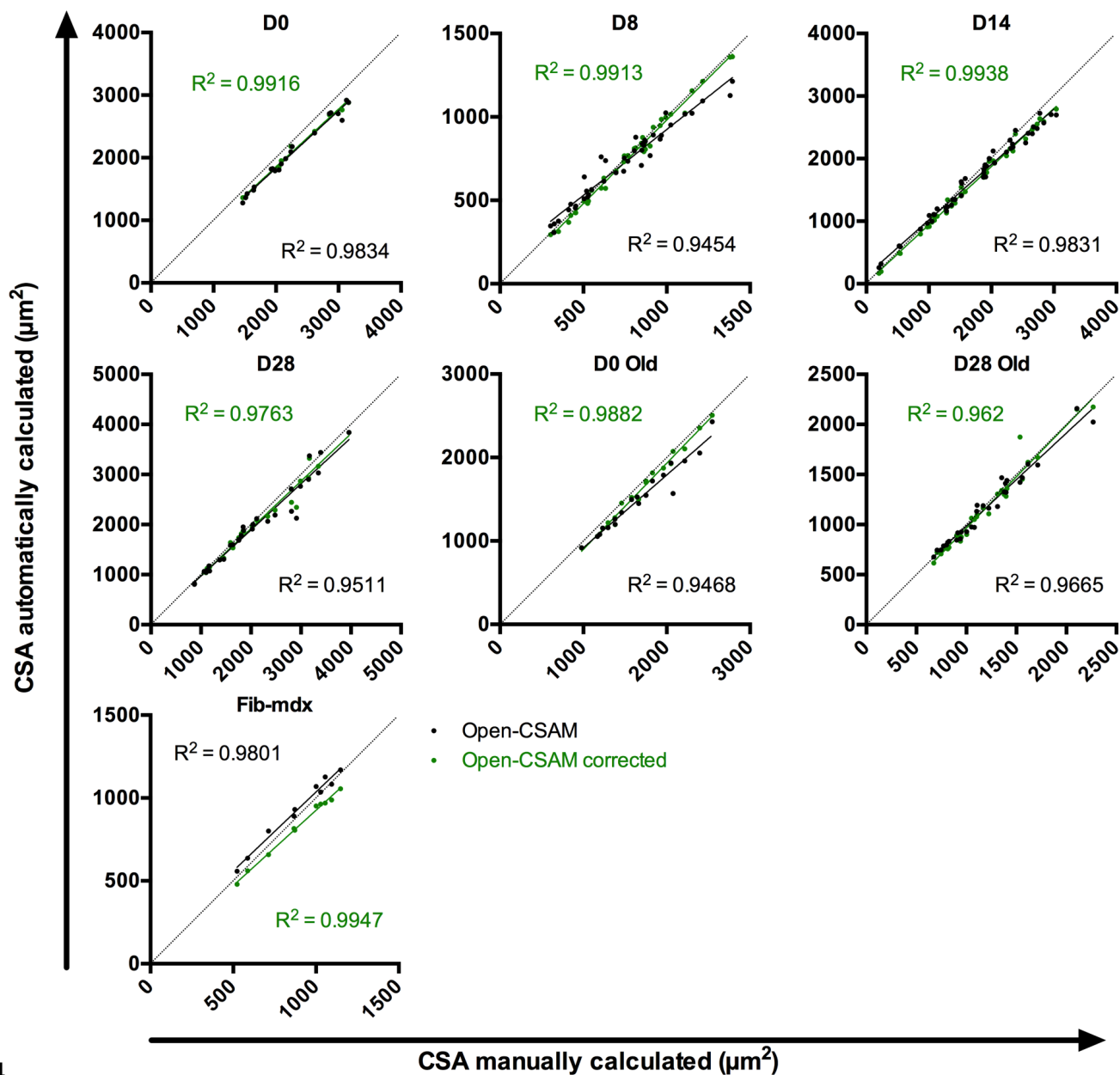

Figure S4

Supplement: Supplementary file 4 — Figure S4. Mean CSA was measured manually and with Open-CSAM, MyoVision, MuscleJ, or SMASH softwares on the same samples as described in Fig. 2a. Muscles were isolated from 8- to 12-week-old mice 8 days (D8), 14 days (D14), and 28 days (D28) post-CTX injury, from uninjured (D0 old) or 28 days post-CTX injury (D28 old) 2-year-old mice. A The correlation between manual measurement (X axis) and Open-CSAM (without manual correction), MyoVision, MuscleJ, or SMASH (Y axis) measurements is presented. B Correlation between manual measurement (X axis) and Open-CSAM (Y axis) before and after manual correction. Each dot represents a picture. The dotted line represents the identity line. (PDF 833 kb) [file 13395_2018_186_MOESM4_ESM.pdf]

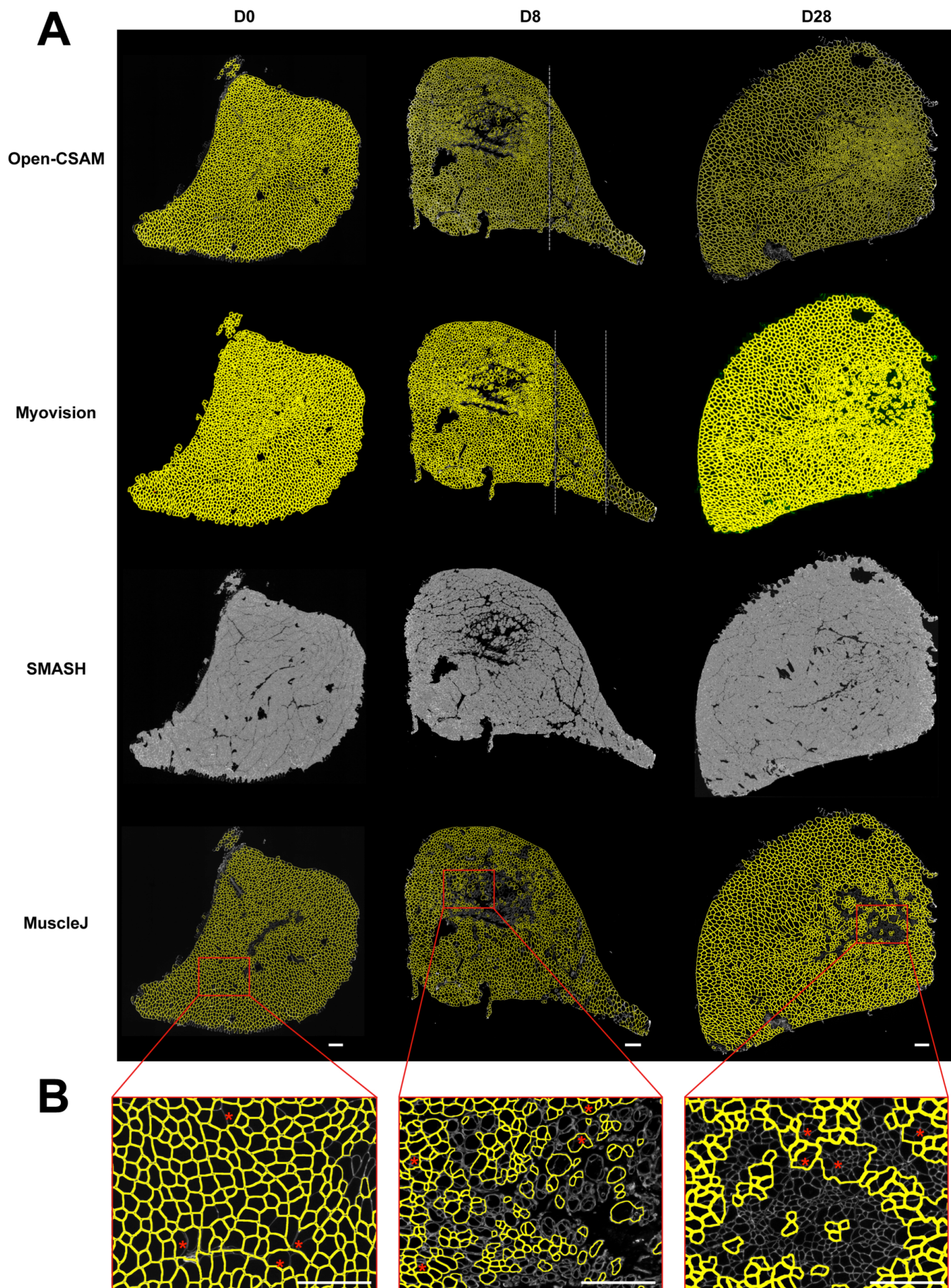

Figure S5

Supplement: Supplementary file 5 — Figure S5. CSA was measured using Open-CSAM, MyoVision, MuscleJ, or SMASH softwares on whole TA muscle images obtained from uninjured (D0) or 8 days (D8) and 28 days (D28) post-CTX injury. A Pictures showing the myofibers (yellow shapes except for SMASH which is in gray) detected by Open-CSAM, MyoVision, SMASH, and MuscleJ. The white dotted lines show where the images were split for Open-CSAM and MyoVision analysis. B Red boxes represent zoom-in examples of specific areas obtained by MuscleJ. Red asterisks show examples of group of myofibers that are merged. White bar = 250 μm. (PDF 12792 kb) [file 13395_2018_186_MOESM5_ESM.pdf]
